# Prediction of Outcome After Endovascular Embolectomy in Anterior Circulation Stroke Using Biomarkers

**DOI:** 10.1007/s12975-021-00905-5

**Published:** 2021-03-15

**Authors:** Fani Pujol-Calderón, Henrik Zetterberg, Erik Portelius, Pia Löwhagen Hendén, Alexandros Rentzos, Jan-Erik Karlsson, Kina Höglund, Kaj Blennow, Lars E. Rosengren

**Affiliations:** 1 Department of Psychiatry & Neurochemistry, University of Gothenburg, Gothenburg, Sweden; 2 Clinical Neurochemistry Laboratory, Sahlgrenska University Hospital, Mölndal, Sweden; 3 Department of Neurodegenerative Disease, UCL Institute of Neurology, Queen Square, London, UK; 4 UK Dementia Research Institute at UCL, London, UK; 5 Department of Anesthesiology and Intensive Care Medicine, University of Gothenburg, Gothenburg, Sweden; 6 Department of Radiology, University of Gothenburg, Gothenburg, Sweden; 7 Department of Neurology, University of Gothenburg, Gothenburg, Sweden

**Keywords:** Stroke, Biomarker, Endovascular treatment, Prediction of outcome

## Abstract

Stroke is a major public health problem that can cause a long-term disability or death due to brain damage. Serious stroke is frequently caused by a large vessel occlusion in the anterior circulation, which should be treated by endovascular embolectomy if possible. In this study, we investigated the use of the brain damage biomarkers tau, NFL, NSE, GFAp, and S100B to understand the progression of nervous tissue damage and their relationship to outcome in such stroke after endovascular treatment. Blood samples were taken from 90 patients pre-treatment and 2 h, 24 h, 48 h, 72 h and 3 months after endovascular treatment. Stroke-related neurological deficit was estimated using the National Institute of Health Stroke Scale (NIHSS) at admission and at 24 h. Neurological outcome was evaluated at 3 months. After stroke, tau, NFL, GFAp and S100B increased in a time dependent manner, while NSE remained constant over time. At 3 months, tau and GFAp levels were back to normal whereas NFL was still high. Tau, NFL and GFAp correlated well to outcome, as well as to infarct volume and NIHSS at 24 h. The best time for prediction of poor outcome was different for each biomarker. However, the combination of NIHSS at 24 h with either tau, NFL or GFAp at 48 h gave the best prediction. The use of biomarkers in the early setting after endovascular treatment of stroke will lead to a simplified and standardized way to estimate the nervous tissue damage and possibly complement the clinical judgement in foreseeing the need of rehabilitation measures.

**Supplementary Information:**

The online version contains supplementary material available at 10.1007/s12975-021-00905-5.

## Introduction

Ischaemic stroke was ranked as the second most common cause of death in the Global Burden of Diseases, Injuries, and Risk Factors Study (GBD 2016). Globally in 2016, there were 80.1 million prevalent stroke cases from which 13.7 million were new cases. Of the total prevalent cases, 84.4% were ischaemic [1]. Hence, stroke is a major public health problem causing long-term disability, lasting brain damage or even death. Stroke caused by large vessel occlusion in the anterior circulation causing moderate to severe stroke is associated with poor outcome if untreated. It is the underlying cause of at around a tenth of all ischaemic stroke [2, 3]. However, endovascular embolectomy combined with intravenous thrombolysis provides an efficient evidence-based treatment [4] and should if possible be used for these patients.

After an ischaemic stroke, cells start to die *within minutes* and parts of the brain become damaged. Necrotic tissue releases neuronal and glial proteins into the cerebrospinal fluid (CSF) and blood. These proteins can be used as biomarkers to determine the degree of damage, as indicators of disease prognosis, as well as to predict and monitor the response to an intervention [5–7].

Tau is a microtubule-associated protein, mainly located in unmyelinated axons [8]. Its main function is to stabilize microtubules. By regulating the microtubule assembly, it allows the reorganisation of the cytoskeleton [9]. It has also been reported that tau regulates axonal transport by different mechanisms [10]. Tau has also been detected in dendrites; however, its function there is still unclear [11]. Tau is an established biomarker in neurodegenerative diseases such as Creutzfeldt-Jakob disease and Alzheimer’s disease [12, 13] and is believed to reflect ongoing axonal degeneration. Levels of tau are increased in both blood and CSF after stroke [5, 13]. Tau immunoreactivity has been shown to be decreased in rodent brain in the infarcted region 24 h after experimental large vessel occlusion [14].

Neurofilament light (NFL) is one of the four members of the neurofilament protein family, which is composed of ɑ-internexin (INA), NFL, as well as neurofilament medium and heavy [15]. They are the main components of intermediate filaments in the brain [15, 16], abundant in large and myelinated axons where they assemble into intermediate filaments to provide structure and stability determining the axonal calibre [17]. NFL was first described in CSF as a marker in neurodegenerative disease but also after stroke [18]. NFL immunoreactivity, contrary to that of tau, has been shown not to be decreased in rodent brain in the infarcted region 24 h after experimental large vessel occlusion [19]. High CSF NFL is found after acute damage to the brain such as in stroke, traumatic brain injury and subarachnoid haemorrhage, as well as in several neurodegenerative diseases [5, 20–22] and it is reported to increase with age [23].

Neuron-specific enolase (NSE) is an isozyme of the glycolytic enzyme enolase [24]. Human NSE is a major brain protein that constitutes between 0.4% and 2.2% of the total soluble protein of the brain, depending on the region [25] making it a plausible marker of neurons [26], but NSE is also expressed in neuroendocrine tissue, erythrocytes and platelets [27, 28]. NSE has been proposed as a biomarker for neuronal damage in traumatic brain injury, stroke as well as a tool in cancer diagnostics [29–31]. Blood NSE dynamics after stroke are controversial, while some studies show an increase of NSE [32] others report no significant changes over time [33, 34].

Glial fibrillary acidic protein (GFAp) is the main intermediate filament protein in astrocytes [35]. GFAp is a vital component of the astroglial cytoskeleton providing mechanical strength to the cell [36, 37]. It also has a number of other functions such as playing a role in suppressing neuronal proliferation in the mature brain [38], forming a physical barrier to isolate damaged tissue [39, 40], as well as regulating the blood flow following ischemia [41]. GFAp immunoreactivity has been shown to be decreased in infarcted regions of post-mortem human brain [42]. CSF levels of GFAp are increased in neurodegenerative diseases such as AD and multiple sclerosis [43, 44] as well as after stroke [45, 46] and traumatic brain injury [47]. GFAp has also been reported to increase with age [23].

S100B is one of the 20 proteins that belong to the S100 protein family; they represent the largest subgroup of Ca^2+^-binding proteins characterized by the EF-hand structural motif [48]. In the nervous system, S100B is mainly found in astrocytes but also in other cell types and its presence is not restricted to neuronal tissue as it is expressed e.g. in adipose tissue [49, 50]. S100B has been reported to increase in the CSF and blood after stoke [46] but also in other acute disorders such as traumatic brain injury [51].

In this study, we investigated the performance of the blood biomarkers tau, NFL, NSE, GFAp and S100B in comparison and combination with clinical biomarkers at different time points after acute ischaemic stroke. This novel study design allowed us to examine the relationship of tau, NFL, NSE, GFA and S100B to clinical outcome and determine their potential use as prognostic biomarkers as well as to further understand the progression of nervous tissue damage after endovascular treatment of acute ischaemic stroke due to large vessel occlusion.

## Methods

### Samples

The samples used in this study are all from the participants in the AnStroke trial described by Löwhagen Hendén et al. 2017 [52]. The AnStroke trial was designed to evaluate the effect of endovascular treatment in patients with large vessel occlusions in the anterior supratentorial circulation carried out in patients receiving general anaesthesia compared to patients receiving conscious sedation. In short, the patients were 18 years or older, with a proven occlusion in anterior cerebral circulation as shown by computed tomographic (CT) angiography, and an admission National Institute of Health Stroke Scale (NIHSS) score ≥10 (if right-sided occlusion) or ≥14 (if left-sided occlusion). After informed consent, patients were randomly distributed in two groups to receive either general anaesthesia (GA) or conscious sedation (CS) in a 1:1 ratio during endovascular treatment which was initiated within 8 h after onset of symptoms. The study included 90 patients.

Ethical approval for the study was obtained from Gothenburg Regional Ethical Review Board (Dnr 013-13).

The blood was collected longitudinally from all 90 patients (the AnStroke cohort) before endovascular treatment (pre) and at intervals after the procedure: 2 h, 24 h, 48 h, 72 h, and 3 months. The median age of the cohort was 72 years (65–80 years) and 54% were men.

### Clinical Biomarkers

Clinical stroke severity at admission and at 24 h was estimated using the NIHSS. To assess early ischaemic changes before intervention, the Alberta Stroke Program Early CT (ASPECT) score was used [53]. The angiographic degree of recanalization after the embolectomy was defined according to the modified thrombolysis in cerebral ischaemia (mTICI) score 0 to 3 (0–2a, not successful recanalization; 2b-–3, successful recanalization). The volume of the infarcted tissue was calculated with a noncontrast CT on day 1 (22 to 36 h) and with magnetic resonance imaging (MRI) on day 3 (2 to 4 days) after treatment. Henceforth, these parameters are referred to as clinical biomarkers in this article. The degree of disability or dependence in the daily activities was assessed by modified ranking scale (mRS) 3 months after stroke and is referred as the outcome in the rest of the study.

### Blood Biomarkers

Tau was measured in plasma with the tau Simoa 2.0 assay (Quanterix) according to manufacturer’s instructions. NFL was analysed in plasma with the NFL ELISA kit from UmanDiagnostics (NF-light® ELISA kit, UmanDiagnostics AB) transferred onto the Simoa platform using a homebrew kit (Quanterix) as previously described [54]. GFAp was determined in serum using the Simoa GFAp kit (Quanterix) according to manufacturer’s instructions. The concentrations of NSE and S100B were measured in serum using immunoassays with electrochemiluminescence detection on the Elecsys platform (Roche Diagnostics) according to manufacturer’s instructions.

### Statistics

Mixed effect models were used to evaluate the change in the biomarker concentrations over time. Subjects were included as random factors and age, gender, NIHSS at admission, type of anaesthesia, the use or no use of intravenous thrombolysis prior to procedure, the time from onset to endovascular treatment and the use or no use of vasoactive drug during the embolectomy procedure as covariates. The addition of NIHSS at admission as a covariate was to count for the possibility that patients with a more severe injury may have higher biomarker concentrations. The addition of type of anaesthesia was to find out whether type of anaesthesia has an impact on neurological outcome. As for the inclusion of the use or no use of intravenous thrombolysis and vasoactive drug (dopamine, ephedrine, phenylephrine or norepinephrine, added at the discretion of the attending anaesthesiologist) as well as the time of onset to endovascular treatment was because of their possible impact on the release of the biomarkers in the blood stream. Spearman’s non-parametric test was used to calculate the correlations between groups (*r*_*s*_). Due to the main interest of comparing the performance of blood biomarkers to clinical biomarkers, correlations between these two groups were the only ones performed. Receiver operating characteristic (ROC) analysis was performed to predict the unfavourable outcome of ischaemic stroke patients (mRS 3-6) based on the biomarker level and the clinical income parameters. Sensitivities, specificities, positive predicted values (PPV) and negative predicted values (NPV) were obtained as measures of performance for the time points with the best area under the curve (AUC). The optimal threshold for the ROC curves was calculated by Youden’s index using the maximum summation of the sensitivity plus specificity of the mRS≤2 vs mRS≥3 groups. Binary logistic regression and crosstabs were used to calculate PPV and NPV. Statistical significance was set at *p*≤0.05. Statistical analyses were performed using SPSS software, version 23.0 and Microsoft Excel (2016) was used to calculate Youden’s index.

## Results

### Blood Biomarker Progression

**Fig. 1:**
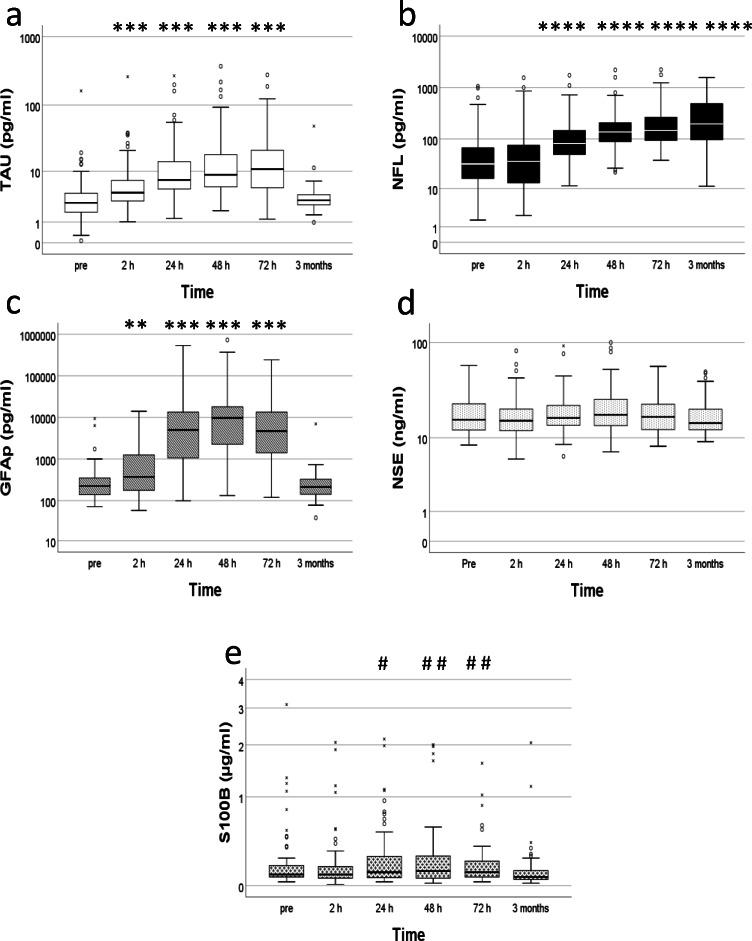
Biomarker progression. Concentration of biomarkers per time point. **a** TAU pg/ml. **b** NFL pg/ml. **c** GFAP pg/ml. **d** NSE ng/ml. **e** S100B μg/ml. °=outlier, x=extreme value. Statistical significance comparing to pre ✱=*p*≤0.05; ✱✱=*p*≤0.01; ✱✱✱=*p*≤0.001; ✱✱✱✱=*p*≤0.0001. (S100B: statistical significance comparing to 3 months #=*p*≤0.05; # #=*p*≤0.01; # # #=*p*≤0.001; # # # #=*p*≤0.0001)

Each biomarker progressed slightly different over time (Fig. 1). Tau was lowest before embolectomy (median, IQR; 2.8, 1.7–4.2 pg/ml) and then increased over time until 72 h [2h (4.4, 3.1–7.2 pg/ml), 24 h (7.2, 5.0–15 pg/ml), 48 h (8.8, 5.4–18 pg/ml) and 72 h (11, 5.3–23 pg/ml)]. The levels were almost back to pre-concentrations at 3 months (3.2, 2.6–4.2 pg/ml) (Fig. 1a). There were significant associations between tau and time (*p*≤0.001), as well as admission NIHSS (*p*≤0.001) and the use of vasoactive drug during embolectomy (*p*≤0.05). No significant associations between tau and age or gender, type of anaesthesia used during procedure, the use of intravenous thrombolysis prior to procedure or the time from onset to start of treatment were observed.

NFL had a slower increase over time compared to tau (Fig. 1b). The lowest concentrations were found pre-embolectomy (32, 16–67 pg/ml) and then continuously increased during the 3 months period [2 h (36, 13–76 pg/ml), 24 h (81, 48–147 pg/ml), 48 h (137, 87–211 pg/ml), 72 h (147, 94–273 pg/ml) and 3 months (197, 95–495 pg/ml)]. There were significant associations between NFL and age (*p*≤0.0001), as well as gender (*p*≤0.05), men being the ones with higher NFL, and time (*p*≤0.0001). No significant associations between NFL and the use of vasoactive drug during embolectomy or the admission NIHSS, the type of anaesthesia used during procedure, the use of intravenous thrombolysis prior to procedure or the time from onset to start of treatment were observed.

GFAp reached its highest concentration at 48 h [pre (223, 139–347 pg/ml), 2 h (372, 177–1259 pg/ml), 24 h (5012, 1030–13670 pg/ml), 48 h (9753, 2155–18172 pg/ml), 72 h (4710, 1330–13867 pg/ml)]. The levels were back to baseline at 3 months (211, 141–327 pg/ml) (Fig. 1c). There were significant associations between GFAp and time (*p*≤0.001), as well as age (*p*≤0.01), gender (*p*≤0.05), men being the ones with higher GFAp, admission NIHSS (*p*≤0.001), the use of intravenous thrombolysis prior to procedure (*p*≤0.001) and the time from onset to start of treatment (*p*≤0.0001). No significant associations between GFAp and the use of vasoactive drug during embolectomy or the type of anaesthesia used during procedure were observed.

NSE remained constant over time (Fig. 1d). Pre-embolectomy (16, 12–23 ng/ml), 2 h (15, 12–21 ng/ml), 24 h (17, 14–23 ng/ml), 48 h (18, 14–26 ng/ml), 72 h (17, 12–24 ng/ml) and 3 months (15, 12-–21 ng/ml). No statistical association was found between NSE and time, although there was a general association between NSE and age (*p*≤0.05). No significant associations between NSE and gender, or the use of vasoactive drug during embolectomy, the admission NIHSS, the type of anaesthesia used during procedure, the use of intravenous thrombolysis prior to procedure or the time from onset to start of treatment were observed.

S100B, similar to GFAp, had its highest concentration at 48 h [pre (90, 68–170 μg/ml), 2 h (90, 60–160 μg/ml), 24 h (110, 60–270 μg/ml), 48 h (120, 60–270 μg/ml), 72 h (110, 70–215 μg/ml)], and then back to baseline values at 3 months (70, 50–127 μg/ml). Although there was a significant association between time and S100B (*p*≤0.05), there were no statistically significant differences between pre and any of the other time points. However, there were significances between 3 months and 24 h, 48 h and 72 h (Fig. 1e). A significant association between age and S100B (*p*≤0.01) was also found. No significant associations between S100B and gender, or the use of vasoactive drug during embolectomy, the admission NIHSS, the type of anaesthesia used during procedure, the use of intravenous thrombolysis prior to procedure or the time from onset to start of treatment were observed.

### Blood Biomarker Correlation to Volume of Infarct Days 1 and 3

The volume of the infarcted tissue measured at day 1 using CT and at day 3 using MRI correlated to all biomarkers at least at two time points (Table S1-5). Volume day 1 correlated best with tau at 72 h (*r*_*s*_=0.58, *p*≤0.0001), NFL at 3 months (*r*_*s*_=0.54, *p*≤0.0001), GFAp at 48 h (*r*_*s*_=0.71, *p*≤0.0001), NSE at 72 h (*r*_*s*_=0.30, *p*≤0.05) and S100B at 24 h (*r*_*s*_=0.27, *p*≤0.05). Volume day 3 had the highest correlation with tau at 48h (*r*_*s*_=0.66, *p*≤0.0001), NFL at 3 months (*r*_*s*_=0.68, *p*≤0.0001), GFAp at 48 h (*r*_*s*_=0.67, *p*≤0.0001), NSE at 72 h (*r*_*s*_=0.38, *p*≤0.05) and S100B at 48 h (*r*_*s*_=0.30, *p*≤0.05). GFAp was the only marker that already at 2 h correlated significantly with volumes days 1 and 3 (*r*_*s*_=0.51, *p*≤0.0001 and *r*_*s*_=0.47, *p*≤0.0001, respectively).

### Blood Biomarker Correlation to NIHSS

Tau, NFL and GFAp correlated to admission NIHSS at least at one time point with the best correlation at 48 h for all three of them [tau (*r*_*s*_=0.33, *p*≤0.01), NFL (*r*_*s*_=0.33, *p*≤0.01) and GFAp (*r*_*s*_=0.35, *p*≤0.01)]. NSE and S100B which did not correlate to admission NIHSS at any time point (Table S1-5).

All biomarkers correlated to NIHSS at 24h at least at one time point (Table S1-5). Tau correlated best at 48 h (*r*_*s*_=0.48, *p*≤0.0001), NFL at 3 months (*r*_*s*_=0.60, *p*≤0.0001), NSE at 24 h (*r*_*s*_=0.28, *p*≤0.05), GFAp at 72 h (*r*_*s*_=0.62, *p*≤0.0001) and S100B at 3 months (*r*_*s*_=−0.36, *p*≤0.01). Already at 2 h, GFAp correlated significantly with NIHSS at 24h (*r*_*s*_=0.53, *p*≤0.0001).

### Blood Biomarker Correlation to ASPECTS

Tau, NFL and GFAp correlated to admission ASPECTS at least at two time points with the best correlation at 24 h for tau (*r*_*s*_=−0.27, *p*≤0.05), 72 h for NFL (*r*_*s*_=−0.36, *p*≤0.01) and 72h for GFAp (*r*_*s*_=−0.37, *p*≤0.01)]. NSE and S100B did not correlate to admission ASPECTS at any time point (Table S1-5).

All biomarkers correlated to ASPECTS at day 3 at least at two time points (Table S1-5). Tau correlated best at 48 h (*r*_*s*_=−0.61, *p*≤0.0001), NFL at 3 months (*r*_*s*_=−0.55, *p*≤0.0001), NSE at 24 h (*r*_*s*_=−0.37, *p*≤0.01), GFAp at 72 h (*r*_*s*_=−0.56, *p*≤0.0001) and S100B at 24 h (*r*_*s*_=−0.33, *p*≤0.01). GFAp was the only marker that already at 2 h correlated significantly with ASPECTS day 3 (*r*_*s*_=0.51, *p*≤0.0001).

### Blood Biomarker Correlation to mTICI

GFAp correlated weakly to mTICI at two time points, with the best correlation at 72 h (*r*_*s*_=−0.28, *p*≤0.05). NSE correlated weakly to mTICI at 3 months (*r*_*s*_=0.33, *p*≤0.05). Tau, NFL and S100B did not correlate to mTICI at any time point (Table S1-5).

### Blood Biomarker Correlation to Severity of Outcome

All biomarkers correlated to the 3 month mRS at least at one time point (Table S1-5); Tau correlated best at 48 h (*r*_*s*_=0.51, *p*≤0.0001) and NFL at 3 months (*r*_*s*_=0.63, *p*≤0.0001). NSE only had a weak correlation at 24 h (*r*_*s*_=0.28, *p*≤0.05). GFAp had its highest correlation at 72h (*r*_*s*_=0.55, *p*≤0.0001) and S100B at 24 h (*r*_*s*_=0.32, *p*≤0.01). Already at 2 h, both tau and GFAp correlated significantly with 3-month mRS (*r*_*s*_=0.42, *p*≤0.0001 and *r*_*s*_=0.49, *p*≤0.0001, respectively).

### Clinical Biomarker Correlation to Severity of Outcome

All clinical parameters except admission ASPECTS correlated to the 3-month mRS; admission NIHSS (*r*_*s*_=0.31, *p*≤0.01), NIHSS at 24 h (*r*_*s*_=0.62, *p*≤0.0001), ASPECTs at day 3 (*r*_*s*_=−0.41, *p*≤0.0001), infarct volume at day 1 (*r*_*s*_=0.43, *p*≤0.0001), infarct volume at day 3 (*r*_*s*_=0.43, *p*≤0.0001) and mTICI (*r*_*s*_=−0.28, *p*≤0.01). The time from onset to start of treatment had a weak but significant correlation with the severity of outcome at 3 months (*r*_*s*_=0.117, *p*≤0.05).

### Blood Biomarker Prediction of Outcome

**Fig. 2:**
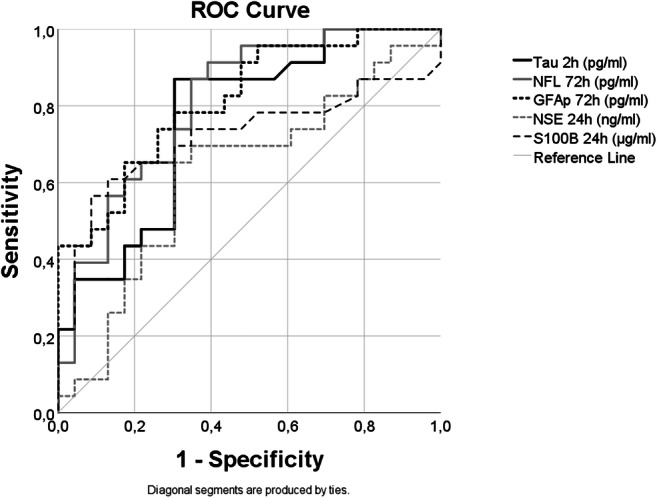
Biomarker prediction of outcome. Receiver operating characteristic (ROC) curves for all biomarkers at the time point where they have the highest area under the curve (AUC) except for NFL. Tau at 2 h (AUC= 0.76), NFL at 72 h (AUC= 0.79), NSE at 24 h (AUC=0.67), GFAP at 72 h (AUC= 0.81) and S100B at 24 h (AUC= 0.70)

The best time points to predict poor outcome, defined as mRS≥3, were for tau at 2 h (AUC of 0.76), 24 h (0.75) and 48 h (0.76) and for GFAp at 72 h (0.81), but also at 2 h (0.76), 24 h (0.72) and 48 h (0.72). NFL had the highest predictive capacity at 72 h (0.79), but also at 24 h (0.76) and 48 h (0.78) (Fig. 2, Table 1) and for NSE at 24 h (0.67) and S100B at 24 h (0.70). Interestingly, NFL had the highest AUC at 3 months (0.88), i.e. at the same time point as the outcome was evaluated. Similarly, the value of GFAp was relatively high (0.75) at this latter time point.

**Table 1:** Blood biomarker prediction of poor outcome (mRS≥3)

	Time	AUC	Cutoff	Sensitivity	Specificity	PPV	NPV
Tau	Pre	0.63	3.2	48.9	68.6	67.6	50
	2 h	0.76	4.03	77.3	73.5	79.1	71.4
	24 h	0.75	6.69	76.1	63.6	74.5	65.6
	48 h	0.76	13	57.9	92.9	91.7	61.9
	72 h	0.67	14.01	51.7	87.5	83.3	60
	3 months	0.59	3.07	66.7	57.6	53.3	70.4
NFL	Pre	0.67	20.55	80.9	51.4	69.1	66.7
	2 h	0.68	37.45	63.6	70.6	73.7	60
	24 h	0.76	53	91.3	60.6	76.4	83.3
	48 h	0.78	149.55	65.8	78.6	80.6	62.9
	72 h	0.79	120.25	86.2	62.5	73.5	78.9
	3 months	0.88	447.95	62.5	97	93.8	78
GFAp	Pre	0.64	140.9	85.1	42.9	66.7	68.2
	2 h	0.76	371.36	72.7	79.4	81.2	69.2
	24 h	0.72	2325.41	78.3	57.6	72	65.5
	48 h	0.79	14163.6	60.5	78.6	79.3	59.5
	72 h	0.81	7280.84	62.1	83.3	81.8	64.5
	3 months	0.75	239.78	66.7	66.7	75.8	75.8
NSE	Pre	0.5	12.43	76.6	34.3	61	52.2
	2 h	0.55	11.87	84.1	36.4	63.8	63.2
	24 h	0.67	15.11	73.3	63.6	73.3	63.6
	48 h	0.59	18.46	59.5	67.9	71	55.9
	72 h	0.55	21.08	41.4	79.2	70.6	52.8
	3 months	0.45	20.4	30.4	81.8	53.8	62.8
S-100	Pre	0.56	0.2	25.5	94.3	85.7	48.5
	2 h	0.59	0.275	20.5	97	90	47.8
	24 h	0.7	0.085	76.1	63.6	74.5	65.6
	48 h	0.62	0.175	52.6	75	74.1	53.8
	72 h	0.55	0.245	24.1	87.5	70	48.8
	3 months	0.39	0.025	100	3	41.8	100

Areas under the curve, cutoff concentration calculated by Youden’s Index (Tau, NFL and GFAp in pg/ml, NSE in ng/ml and S100B in μg/ml), sensitivity (%), specificity (%), positive predicted value (PPV) (%) and negative predicted value (NPV) (%) for each blood biomarker per time point. *mRS* modified ranking scale

### Clinical Biomarker Prediction of Outcome

**Fig. 3:**
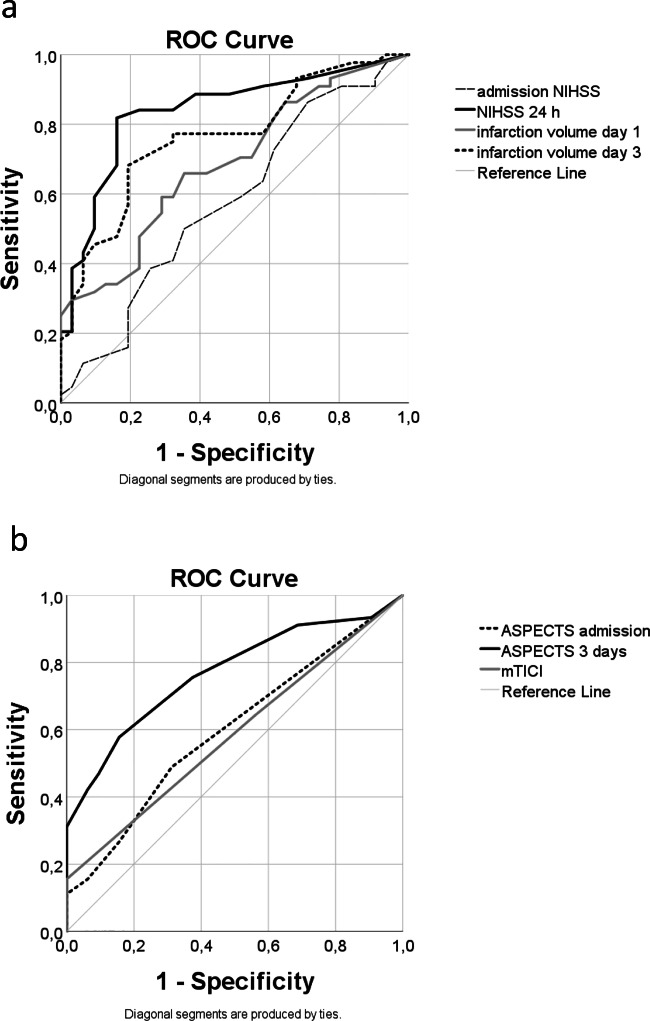
Clinical biomarker prediction of outcome at 3 months (mRS). Receiver operating characteristic curves for the clinical biomarkers. **a** Admission NIHSS (AUC=0.65), NIHSS at 24 h (AUC=0.86), infarct volume on day 1 (AUC=0.73) and infarct volume on day 3 (AUC=0.78). **b** Admission ASPECTS (AUC=0.60), ASPECTS at 3 days (AUC=0.77) and mTICI (AUC=0.61)

Most clinical parameters had a good prediction of poor outcome. Admission NIHSS predicted for poor outcome with an AUC=0.65, NIHSS at 24 h with an AUC=0.86, admission ASPECTS with an AUC=0.60, ASPECTS at 3 days with an AUC=0.77. Infarct volume on day 1 predicted for poor outcome with an AUC=0.73 and infarct volume on day 3 had an AUC=0.78. mTICI predicted for poor outcome with an AUC=0.61 (Fig. 3, Table S6).

### Combination of the Blood and Clinical Biomarker Prediction of Outcome

**Fig. 4:**
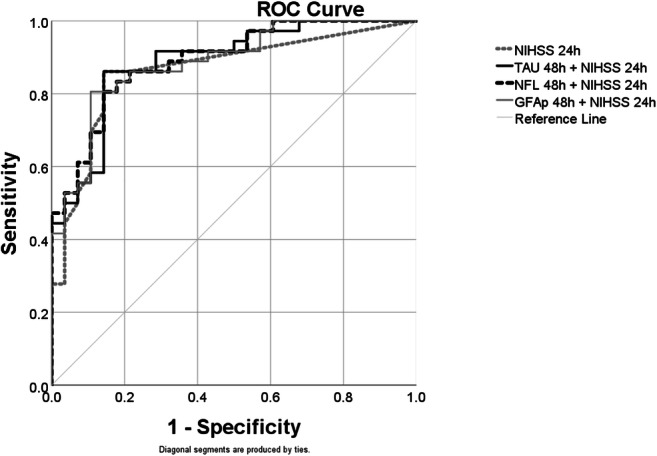
Combination of biomarker prediction of outcome. Receiver operating characteristic (ROC) curves for the combinations of NIHSS at 24 h with Tau, NFL and GFAp at 48 h in comparison with NIHSS at 24 h alone. NIHSS at 24 h (AUC= 0.86), Tau at 48 h + NIHSS at 24 h (AUC= 0.89), NFL at 48 h + NIHSS at 24 h (AUC= 0.89) and GFAP at 48 h + NIHSS at 24 h (AUC= 0.89)

To assess if the prediction for poor outcome could be improved by the combination of clinical parameters and blood biomarkers, we combined the clinically easily accessible parameter NIHSS at 24 h and biomarkers with AUC>0.7 at 48 h, i.e. tau, NFL and GFAp (Fig. 4).

Overall, all the combinations improved the prediction of poor outcome when compared to the prediction with a single factor. All combinations had similar predictive capacity (AUC=0.89) (Table S7).

## Discussion

In the present study, we describe the temporal pattern of tau, NFL, GFAp, NSE and S100B in the blood after stroke following large vessel occlusions and embolectomy in the anterior circulation of the brain. Different kinetics for each biomarker were observed, possibly reflecting their cellular location, considering that tau, NFL and NSE are neuronal markers while GFAp and S100B belong to astroglial cells. Tau, NFL and GFAp are essentially nervous tissue-specific whereas S100B and NSE are present outside the nervous system in, e.g. the adipose and neuroendocrine tissue as well as the blood cells.

In general, tau and GFAp followed an arch-like pattern in response to the stroke-induced brain damage with increasing concentrations the three first days followed by a decrease back to baseline values in the next sample acquired at 3 months. At their max concentration time points, tau (72 h) had increased 4 times and GFAp (48 h) 43 times their baseline concentrations. NFL, on the other hand, continuously kept rising from pre-treatment levels to a 6-fold increase at 3 months. S100B seemingly displayed a time-dependent curve with slightly increased levels at 24 and 48 h; however, not significantly when compared with pre levels. Even so, there was a week and significant correlation to infarct volume at these times. NSE remained constant over time although a weak correlation to infarct volume was seen at 24 and 72 h. Hence, S-100 and NSE do not seem to reflect the underlying focal ischemia induced brain damage as good as NFL, tau and GFAp.

A similar arch pattern has previously been described for tau in CSF and [13] blood [55] after stroke, showing normal concentrations of tau at baseline, increasing about 5 times and maintaining a plateau for the coming weeks before returning back to baseline after 3 months. Tau has also been found increased in blood after stroke in other studies [5, 56].

Similarly, NFL progression after ischaemic stroke has been described in both CSF and serum [57], showing a constant increase up to 3 weeks followed by a decrease at 3 months, though still being very high at this latter time point. The fact that in the present study, we do not have a measurement at around 3 weeks means that we have missed the NFL’s peak and that possibly the observed levels at 3 months are reflecting a start of a decrease, even though it has been shown that NFL is still increased after 12 months when compared to controls [58]. Others have also observed elevated levels in the early phase after stroke, but they did not study such an extended time period [5, 59]. Even so, long sustained increase of CFS NFL with maximal levels within the first month after the acute event followed by a slow decreased over several months has previously been shown after acute brain damage due to herpes simplex virus type 1 encephalitis and exacerbation of multiple sclerosis [60, 61]. Thus, a similar kinetics seems plausible after ischaemic stroke.

GFAp levels have also been reported augmented in longitudinal CSF samples from acute ischaemic stroke patients showing substantially increased levels at days 2–3 after stroke onset compared to levels within 24 h, remaining equally high at 7 days, but then back to initial and normalized levels at 3 weeks and 3 months [62]. In earlier studies serum GFAp has been reported to be below the limit of detection of the ELISAs used [63, 64]. In this study, we used the Simoa platform, to analyse GFAp, a new technique which can, depending on the analyte, be 100 times more sensitive than the conventional ELISA [54], and therefore, we could measure GFAP with better accuracy and at all time points to show its progression pattern.

A previous study showed that both S100B and GFAp in CSF increased similarly in a time and volume dependent manner after ischaemic stroke [46]. Contradictory results have also been observed where a slight increase of S100B on day 3 is followed by a peak on day 6, nevertheless not significant [64]. Even so, it has been advocated that S100B is an unspecific marker due to its tendency to be raised from extracranial sources [65]. A similar reasoning might be possible regarding NSE in blood [28], which in the present study remained at a constant level during the observation period.

In ischaemic stroke, neurons and astrocytes rapidly die in the necrotic infarcted area. The different kinetics of tau, GFAp and NFL observed in the present study may be caused by their cellular and subcellular localisations. GFAp is mainly found in the fibrillary astrocytes of white mater but also in the protoplasmic astrocytes of grey matter [66], and in the present study, this marker responds with a rapid increase probably reflecting the astroglial breakdown in the necrotic tissue. Tau, on the contrary, is a neuronal protein enriched in thin unmyelinated axons of the neuropil of grey matter and less prevalent in myelinated axons [67, 68]. The increase of this marker, probably due to neuronal death, seems to occur at least as early as that of GFAp, imaging the sensitivity of neuronal cell to anoxia. Relatively, levels of tau are higher at 3 days compared to GFAp which tentatively could be caused by a slow neuronal death in the penumbra or secondary axonal degeneration following the degradation of the nerve cell body. On the other hand, the temporal profile of NFL is different with no or only a small increase in early samples followed by higher levels with a maximum observed at 3 months. This extended time course of the NFL increase possibly reflects a post-ischaemic Wallerian degeneration of myelinated axons. Neurofilaments constitute only a minor part of the cytoskeletal constituents in the neuronal cell body and dendrites relative to axons and are abundant in large myelinated axons. The time profile of immunohistochemical as well as radiological findings in Wallerian degeneration after stroke show a delayed and longstanding deteriorating process fitting the release pattern of NFL in the present study [69, 70].

Tau, NFL and GFAp were moderately to highly correlated with the volume of the infarct, the NIHSS at 24 h and to the degree of disability defined by mRS at 3 months. Regarding NSE and S100B however, correlations were very low and mainly insignificant. Shahim et al. showed that S100B in the serum of TBI patients could not differentiate controls from patients, and it did not correlate to outcome [58]. Likewise, Routsi et al. discovered that serum S100B is also elevated in critically ill patients with no obvious brain tissue damage [71]. The role of NSE in stroke is a bit controversial since different studies show opposite results. While some studies have shown that NSE in the blood correlates with volume, stroke severity and its ability to predict for a functional outcome [72, 73]; other studies have shown opposite results [74]. In summary, we conclude that in the present study tau, NFL and GFAp in the blood reflect the extent of nervous tissue damage after ischaemic injury to the brain whereas S100B and NSE are of little value.

Regarding the prediction of outcome according to AUCs, the best performance was seen using NFL at 3 months, but due to the irrelevancy of the time point, we therefore assign NFL at 72 h as the best moment to predict for outcome, followed by GFAp at 72 h and tau at 2 h. NIHSS at 24 h predicted outcome slightly better than the blood biomarkers, whereas ASPECTS at day 3 and volume at both days 1 and 3 predicted outcome at a similar level. NIHSS and ASPECTS at admission and mTICI had a poor prediction for outcome when compared to all the other biomarkers. When NIHSS at 24 h was combined with either NFL, tau or GFAp at 48 h, there was an improvement in the AUCs, sensitivities and specificities for all combinations when compared to single blood biomarkers. When compared to single NIHSS at 24 h, as shown in Fig, 4, AUCs were improved as well as the sensitivity and negative predictive value of its combination with TAU and GFAp. All this taken together suggests that these biomarkers can be used as a complementary test to confirm the severity of lesions in the anterior supratentorial circulation in patients subjected to embolectomy. In the clinical situation, the estimation of NIHSS might not always be of high quality and then objective measures become important. Regarding volume determination, MR at day 3 is generally not routine. Similarly, even if calculation of infarction volume based on CT within 1–2 days is quite possible, the neuroradiologist still generally subjectively estimates size. Levels of tau and GFAp seem to be very early indicator of outcome (2 h) whereas NFL has comparatively delayed kinetics with similar prognostic capability from 24 h, but with a much better association with mRS at 3 months in samples taken simultaneously with the clinical evaluation. Tentatively, NFL at 3 months can be used to complement the clinical evaluation of patient in this later phase after stroke, and possibly prognosticates the future handicap of the patient.

The major strength of this study is that it embodies a common clinical situation in the acute chain of stroke care. Endovascular treatment due to large vessel occlusions is a frequent clinical situation, and in our region, the neurointerventional centre is now treating almost 8% of all ischaemic stroke (year 2019). The size of the study cohort is relatively large (*N*=90), the patients are well characterized including outcome, the samples were taken early followed by several time points and three of the biomarkers (tau, NFL and GFAp) gave interesting information with regard to various clinical parameters, particularly outcome. These biomarkers are well suited to be used as a complement in the clinical setting concerning patient care and rehabilitation following embolectomy in the anterior circulation of the brain due to stroke.

The study also has some limitations. It concerns large infarctions in the supratentorial anterior circulation. No certain conclusions can be drawn regarding minor stroke or stroke in the posterior circulation and the association between these biomarkers and the various clinical parameters. In addition, no assumptions regarding the sensitivity to detect smaller lesions can be made. Moreover, all patients were subjected to an efficient evidence-based treatment and the native course of the disease was for obvious reasons not studied. Finally, the neuroradiological imagining does not allow any conclusions about the extent of grey matter contra white matter involvement.

In summary, this study shows that tau, NFL and GFAp are good candidate biomarkers with different kinetics that reflect the ischaemic brain injury due to large vessel occlusions in the anterior circulation after endovascular treatment and that they can predict the degree of disability 3 months after the stroke. Their use in the early setting after endovascular treatment of stroke could lead to a simplified and standardized way to estimate the nervous tissue damage and possibly complement the clinical judgment in foreseeing the need of rehabilitation measures. For a wider understanding of the potential of these markers, further studies in different types of stroke are needed.

## Supplementary Information

ESM 1 (DOCX 35 kb)

## Data Availability

This article is based on the work included in the doctoral thesis “Neurofilaments as biomarkers of neuronal damage” by Fani Pujol-Calderón.
