# Supplementary material for: Prediction of Outcome After Endovascular Embolectomy in Anterior Circulation Stroke Using Biomarkers
Source: Transl Stroke Res. 2021 Mar 15;13(1):65–76. doi: 10.1007/s12975-021-00905-5 (PMC8766380; doi:10.1007/s12975-021-00905-5)
Supplement: Supplementary file 1 — (DOCX 35 kb) [file 12975_2021_905_MOESM1_ESM.docx]

**Prediction of outcome after endovascular embolectomy in anterior circulation stroke using biomarkers.**

Fani Pujol-Calderón^1*^, Henrik Zetterberg^1,2,3,4^, Erik Portelius^1,2^, Pia Löwhagen Hendén^5^, Alexandros Rentzos^6^, Jan-Erik Karlsson^7^, Kina Höglund^1,2^, Kaj Blennow^1,2^, Lars E. Rosengren^7^

^1^ Department of Psychiatry & Neurochemistry, University of Gothenburg, Sweden

^2^ Clinical Neurochemistry Laboratory, Sahlgrenska University Hospital, Mölndal, Sweden

^3^ Department of Neurodegenerative Disease, UCL Institute of Neurology, Queen Square, London, United Kingdom

^4^ UK Dementia Research Institute at UCL, London, United Kingdom

^5^ Department of Anesthesiology and Intensive Care Medicine, University of Gothenburg, Sweden

^6^ Department of Radiology, University of Gothenburg, Sweden

^7^ Department of Neurology, University of Gothenburg, Sweden

*Corresponding author: fani.pujol.calderon@gu.se

**Table S1. Tau correlations to clinical biomarkers.**

|  |  | Correlation to | | | | | | | |
| --- | --- | --- | --- | --- | --- | --- | --- | --- | --- |
|  |  | Volume  Day 1 | Volume  Day 3 | NIHSS  adm. | NIHSS  24h | ASPECTS  adm. | ASPECTS  Day 3 | mTICI | mRS |
|  | Concentration (pg/ml)  IQR | r_s_  p | r_s_  p | r_s_  p | r_s_  p | r_s_  p | r_s_  p | r_s_  p | r_s_  p |
| pre | 2.8  1.7-4.2 | 0.072  ns | 0.026  ns | 0.06  ns | 0.19  ns | - 0.12  ns | 0.007  ns | -0.11  ns | 0.22  ≤0.05 |
| 2h | 4.4  3.1-7.2 | 0.16  ns | 0.19  ns | 0.22  ns | 0.27  ≤0.05 | - 0.24  ≤0.05 | -0.15  ns | -0.12  ns | 0.42  ≤0.0001 |
| 24h | 7.2  5,0-15 | 0.53  ≤0.0001 | 0.54  ≤0.0001 | 0.24  ≤0.05 | 0.40  ≤0.0001 | - 0.27  ≤0.05 | -0.47  ≤0.0001 | -0.15  ns | 0.44  ≤0.0001 |
| 48h | 8.8  5.4-18 | 0.58  ≤0.0001 | 0.66  ≤0.0001 | 0.33  ≤0.01 | 0.48  ≤0.0001 | - 0.15  ns | -0.61  ≤0.0001 | -0.08  ns | 0.51  ≤0.0001 |
| 72h | 11  5.3-23 | 0.58  ≤0.0001 | 0.65  ≤0.0001 | 0.12  ns | 0.33  ≤0.05 | -0.26  ns | -0.58  ≤0.0001 | -0.14  ns | 0.37  ≤0.01 |
| 3m | 3.2  2.6-4.2 | 0.0  ns | 0.065  ns | 0.13  ns | 0.11  ns | - 0.016  ns | -0.048  ns | -0.047  ns | 0.24  ns |

Tau correlations to clinical biomarkers per time point. r_s_=Spearman correlation coefficient, IQR= inter quartile range, adm.=admission.

**Table S2. NFL correlations to clinical biomarkers.**

|  |  | Correlation to | | | | | | | |
| --- | --- | --- | --- | --- | --- | --- | --- | --- | --- |
|  |  | Volume  Day 1 | Volume  Day 3 | NIHSS  adm. | NIHSS  24h | ASPECTS  adm. | ASPECTS  Day 3 | mTICI | mRS |
|  | Concentration (pg/ml)  IQR | r_s_  p | r_s_  p | r_s_  p | r_s_  p | r_s_  p | r_s_  p | r_s_  p | r_s_  p |
| pre | 32  16-67 | -0.077  ns | -0.022  ns | 0.21  ns | 0.13  ns | - 0.20  ns | 0.025  ns | -0.092  ns | 0.27  ≤0.05 |
| 2h | 36  13-76 | 0.029  ns | 0.099  ns | 0.27  ≤0.05 | 0.18  ns | - 0.27  ≤0.05 | -0.089  ns | -0.14  ns | 0.31  ≤0.01 |
| 24h | 81  48-147 | 0.38  ≤0.001 | 0.41  ≤0.0001 | 0.191  ns | 0.35  ≤0.01 | -0.34  ≤0.01 | -0.39  ≤0.001 | -0.16  ns | 0.49  ≤0.0001 |
| 48h | 137  87-211 | 0.51  ≤0.0001 | 0.55  ≤0.0001 | 0.33  ≤0.01 | 0.43  ≤0.0001 | -0.28  ≤0.05 | -0.53  ≤0.0001 | -0.16  ns | 0.55  ≤0.0001 |
| 72h | 147  97-273 | 0.53  ≤0.0001 | 0.59  ≤0.0001 | 0.23  ns | 0.46  ≤0.0001 | -0.36  ≤0.01 | -0.50  ≤0.0001 | -0.23  ns | 0.55  ≤0.0001 |
| 3m | 197  95-495 | 0.54  ≤0.0001 | 0.68  ≤0.0001 | 0.26  ns | 0.60  ≤0.0001 | - 0.14  ns | -0.55  ≤0.0001 | -0.089  ns | 0.63  ≤0.0001 |

NFL correlations to clinical biomarkers per time point. r_s_=Spearman correlation coefficient, IQR= inter quartile range, adm.=admission.

**Table S3. GFAP correlations to clinical biomarkers.**

|  |  | Correlation to | | | | | | | |
| --- | --- | --- | --- | --- | --- | --- | --- | --- | --- |
|  |  | Volume  Day 1 | Volume  Day 3 | NIHSS  adm. | NIHSS  24h | ASPECTS  adm. | ASPECTS  Day 3 | mTICI | mRS |
|  | Concentration (pg/ml)  IQR | r_s_  p | r_s_  p | r_s_  p | r_s_  p | r_s_  p | r_s_  p | r_s_  p | r_s_  p |
| pre | 223  139-347 | -0.045  ns | -0.007  ns | 0.16  ns | 0.20  ns | -0.048  ns | -0.034  ns | -0.23  ≤0.05 | 0.24  ≤0.05 |
| 2h | 372  177-1259 | 0.51  ≤0.0001 | 0.47  ≤0.0001 | 0.21  ns | 0.53  ≤0.0001 | -0.26  ≤0.05 | -0.51  ≤0.0001 | -0.12  ns | 0.49  ≤0.0001 |
| 24h | 5012  1030-13670 | 0.65  ≤0.0001 | 0.59  ≤0.0001 | 0.23  ≤0.05 | 0.53  ≤0.0001 | -0.32  ≤0.01 | -0.56  ≤0.0001 | -0.12  ns | 0.36  ≤0.001 |
| 48h | 9753  2155-18172 | 0.71  ≤0.0001 | 0.67  ≤0.0001 | 0.35  ≤0.01 | 0.59  ≤0.0001 | -0.26  ≤0.05 | -0.55  ≤0.0001 | -0.22  ns | 0.53  ≤0.0001 |
| 72h | 4710  1330-13867 | 0.68  ≤0.0001 | 0.65  ≤0.0001 | 0.28  ≤0.05 | 0.62  ≤0.0001 | -0.37  ≤0.01 | -0.56  ≤0.0001 | -0.28  ≤0.05 | 0.55  ≤0.0001 |
| 3m | 211  141-327 | 0.26  ns | 0.38  ≤0.01 | 0.13  ns | 0.25  ns | -0.17  ns | -0.35  ≤0.05 | 0.047  ns | 0.37  ≤0.01 |

GFAP correlations to clinical biomarkers per time point. r_s_=Spearman correlation coefficient, IQR= inter quartile range, adm.=admission.

**Table S4. S100B correlations to clinical biomarkers.**

|  |  | Correlation to | | | | | | | |
| --- | --- | --- | --- | --- | --- | --- | --- | --- | --- |
|  |  | Volume  Day 1 | Volume  Day 3 | NIHSS  adm. | NIHSS  24h | ASPECTS  adm. | ASPECTS  Day 3 | mTICI | mRS |
|  | Concentration (µg/ml)  IQR | r_s_  p | r_s_  p | r_s_  p | r_s_  p | r_s_  p | r_s_  p | r_s_  p | r_s_  p |
| pre | 90  68-170 | -0.016  ns | -0.069  ns | 0.18  ns | -0.07  ns | -0.15  ns | -0.07  ns | 0.021  ns | 0.15  ns |
| 2h | 90  60-160 | 0.049  ns | 0.072  ns | 0.11  ns | -0.02  ns | -0.22  ns | -0.083  ns | 0.18  ns | 0.23  ≤0.05 |
| 24h | 110  60-270 | 0.27  ≤0.05 | 0.28  ≤0.05 | 0.22  ns | 0.31  ≤0.01 | -0.13  ns | -0.33  ≤0.01 | -0.009  ns | 0.32  ≤0.01 |
| 48h | 120  60-270 | 0.25  ≤0.05 | 0.30  ≤0.05 | 0.13  ns | 0.21  ns | -0.13  ns | -0.28  ≤0.05 | 0.05  ns | 0.22  ns |
| 72h | 110  70-215 | 0.15  ns | 0.15  ns | -0.069  ns | -0.035  ns | -0.15  ns | -0.14  ns | 0.13  ns | 0.12  ns |
| 3m | 70  50-127 | -0.26  ns | -0.27  ns | 0.020  ns | -0.36  ≤0.01 | 0.044  ns | 0.25  ns | 0.25  ns | -0.13  ns |

S100B correlations to clinical biomarkers per time point. r_s_=Spearman correlation coefficient, IQR= inter quartile range, adm.=admission.

**Table S5. NSE correlations to clinical biomarkers.**

|  |  | Correlation to | | | | | | | |
| --- | --- | --- | --- | --- | --- | --- | --- | --- | --- |
|  |  | Volume  Day 1 | Volume  Day 3 | NIHSS  adm. | NIHSS  24h | ASPECTS  adm. | ASPECTS  Day 3 | mTICI | mRS |
|  | Concentration (ng/ml)  IQR | r_s_  p | r_s_  p | r_s_  p | r_s_  p | r_s_  p | r_s_  p | r_s_  p | r_s_  p |
| pre | 16  12-23 | -0.1  ns | -0.12  ns | 0.046  ns | -0.106  ns | 0.079  ns | 0.16  ns | -0.079  ns | 0.07  ns |
| 2h | 15  12-21 | -0.021  ns | 0.046  ns | 0.1  ns | -0.13  ns | 0.083  ns | -0.032  ns | 0.17  ns | 0.11  ns |
| 24h | 17  14-23 | 0.29  ≤0.05 | 0.30  ≤0.05 | 0.18  ns | 0.28  ≤0.05 | -0.22  ns | -0.37  ≤0.01 | -0.004  ns | 0.28  ≤0.05 |
| 48h | 18  14-26 | 0.23  ns | 0.26  ns | 0.14  ns | 0.098  ns | -0.15  ns | -0.30  ≤0.05 | -0.020  ns | 0.11  ns |
| 72h | 17  12-24 | 0.30  ≤0.05 | 0.38  ≤0.05 | - 0.053  ns | 0.014  ns | -0.18  ns | -0.33  ≤0.05 | 0.016  ns | 0.009  ns |
| 3m | 15  12-21 | -0.24  ns | -0.16  ns | - 0.074  ns | -0.24  ns | 0.14  ns | 0.16  ns | 0.33  ≤0.05 | -0.24  ns |

NSE correlations to clinical biomarkers per time point. r_s_=Spearman correlation coefficient, IQR= inter quartile range, adm.=admission.

**Table S6.** **Clinical biomarkers prediction of outcome.**

|  | NIHSS | NIHSS | ASPECTS | ASPECTS | Volume | Volume | mTICI |
| --- | --- | --- | --- | --- | --- | --- | --- |
| Time | admission | 24h | admission | Day 3 | Day 1 | Day 3 |  |
| AUC | 0.65 | 0.86 | 0.60 | 0.77 | 0.73 | 0.78 | 0.61 |
| cutoff | 18.5 | 7.5 | 9.5 | 5.5 | 17.5 | 22.5 | 2.5 |
| sensitivity | 56.6 | 82.4 | 50.9 | 57.8 | 69.2 | 68.9 | 17 |
| specificity | 67.6 | 86.5 | 67.6 | 81.3 | 66.7 | 81.3 | 100 |
| PPV | 71.4 | 89.4 | 69.2 | 81.3 | 75 | 83.8 | 100 |
| NPV | 52.1 | 78 | 49 | 57.8 | 60 | 65 | 45.7 |

Areas under the curve, cut off values (NIHSS, ASPECTS and mTICI in scores and volumes in ml) calculated by Youden’s Index, sensitivity, specificity, positive predicted value (PPV) and negative predicted value (NPV) for each clinical biomarker.

**Table S7. Combination of clinical and blood biomarkers.**

|  | TAU 48h | NFL 48h | GFAP 48h |
| --- | --- | --- | --- |
|  | + | + | + |
|  | NIHSS 24h | NIHSS 24h | NIHSS 24h |
| AUC | 0.891 | 0.894 | 0.890 |
| sensitivity | 86.5 | 81.8 | 86.5 |
| specificity | 85.7 | 82.1 | 78.6 |
| PPV | 88.9 | 85.7 | 84.2 |
| NPV | 82.8 | 76.7 | 81.5 |

Areas under the curve, sensitivity (%), specificity (%), positive predicted value (PPV) (%) and negative predicted value (NPV) (%) for each biomarker combination.
